# PEDOT/CNT Flexible MEAs Reveal New Insights into the *Clock* Gene's Role in Dopamine Dynamics

**DOI:** 10.1002/advs.202308212

**Published:** 2024-03-02

**Authors:** Bingchen Wu, Elisa Castagnola, Colleen A. McClung, Xinyan Tracy Cui

**Affiliations:** ^1^ Department of Bioengineering University of Pittsburgh Pittsburgh PA 15213 USA; ^2^ Center for the Neural Basis of Cognition Pittsburgh PA 15213 USA; ^3^ Department of Biomedical Engineering Louisiana Tech University Ruston LA 71272 USA; ^4^ Department of Psychiatry University of Pittsburgh Pittsburgh PA 15213 USA; ^5^ McGowan Institute for Regenerative Medicine Pittsburgh PA 15219 USA

**Keywords:** chronic dopamine sensing, chronic electrophysiology, circadian rhythm, flexible MEAs, PEDOT/CNT

## Abstract

Substantial evidence has shown that the *Circadian Locomotor Output Cycles Kaput* (*Clock*) gene is a core transcription factor of circadian rhythms that regulates dopamine (DA) synthesis. To shed light on the mechanism of this interaction, flexible multielectrode arrays (MEAs) are developed that can measure both DA concentrations and electrophysiology chronically. The dual functionality is enabled by conducting polymer PEDOT doped with acid‐functionalized carbon nanotubes (CNT). The PEDOT/CNT microelectrode coating maintained stable electrochemical impedance and DA detection by square wave voltammetry for 4 weeks in vitro. When implanted in wild‐type (WT) and *Clock* mutation (MU) mice, MEAs measured tonic DA concentration and extracellular neural activity with high spatial and temporal resolution for 4 weeks. A diurnal change of DA concentration in WT is observed, but not in MU, and a higher basal DA concentration and stronger cocaine‐induced DA increase in MU. Meanwhile, striatal neuronal firing rate is found to be positively correlated with DA concentration in both animal groups. These findings offer new insights into DA dynamics in the context of circadian rhythm regulation, and the chronically reliable performance and dual measurement capability of this technology hold great potential for a broad range of neuroscience research.

## Graphical abstract

Scalable and reproducible fabrication of flexible MEAs for dopamine and electrophysiology measurements are demonstrated. PEDOT/CNT coated MEAs can stably monitor dopamine levels and electrophysiology for 4 weeks in vivo. Using the *ClockΔ19* mutant mice model, new insights are gained on the *Clock* gene's role in dopamine dynamics. This technique can help better understand the mechanisms of neurological processes and dysfunctions.

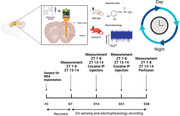

## 1 Introduction

The information exchange among different brain regions is carried out via two types of signaling mechanisms: electric signals (e.g. action potentials) and chemical signals (eg. neurotransmitters).^[^ ^1^ ^]^ The coordinated actions from action potentials and neurotransmitters with complex temporal and spatial patterns eventually manifest into physiological behaviors and high‐level cognitive functions. Among all the functionally diverse neural network systems, the mesolimbic dopaminergic (ML‐DA) system plays an instrumental role in psychological processes and neuropsychiatric diseases.^[^ ^2^, ^3^, ^4^, ^5^, ^6^ ^]^ The ML‐DA system consists of a set of interconnected brain regions that regulate a broad range of cognitive functions such as pleasure, learning, and motivation.^[^ ^2^, ^3^, ^4^, ^5^, ^6^, ^7^, ^8^ ^]^ Thus, a multimodal tool that can measure both DA concentration and neurophysiologic signals in multiple brain regions of the mesolimbic system chronically is highly desired.

Specifically, in the research field of circadian rhythm, substantial evidence has shown that DA is involved in the regulation process of circadian rhythms.^[^ ^8^, ^9^, ^10^, ^11^, ^12^ ^]^ Circadian rhythms—daily rhythms that regulate many behaviors and biological processes—enable organisms to adapt to the environment and optimize behavioral responses for survival. The rhythmic activities are entrained to a variety of stimuli known as zeitgebers (ZT) or “time‐givers” (light, food, etc.).^[^ ^8^ ^]^ The central rhythm‐generating region in the mammalian brain is the suprachiasmatic nucleus (SCN). Subsidiary oscillators are also identified in other brain regions and peripheral tissue that can operate independently or coordinated by SCN.^[^ ^13^ ^]^ The molecular clock machinery that regulates circadian rhythms is present in nearly all cell types throughout the body, consisting of a series of transcriptional‐translational feedback loops. One of the core transcription factors that affect the diurnal regulation of the mammalian circadian rhythms is *Clock*.^[^ ^8^ ^]^ DA has been shown to have a diurnal variation in levels in the striatum; however, the regulation of extracellular DA in striatal regions following a disruption in the *Clock* has not been systematically measured.^[^ ^8^, ^10^, ^11^, ^12^, ^14^, ^15^, ^16^ ^]^ qPCR quantifications of mRNA and protein expression level of tyrosine hydroxylase (TH), the rate‐limiting enzyme for DA production, found an increase in TH expression in the midbrain ventral tegmental area (VTA) in *ClockΔ19* mutant mice (MU), a dominant negative form of *Clock*, compared to the wild type (WT) counterpart.^[^ ^9^ ^]^ Disruption in the *Clock* gene also results in enhanced firing and bursting behaviors of VTA dopaminergic neurons that project to the striatum and release DA.^[^ ^9^, ^17^ ^]^ 95% of striatal neurons are GABAergic medium spiny neurons (MSNs) that differentially express dopamine receptors and are critical in mediating responses to drugs of abuse and rewarding stimuli. Previous work has examined electrophysiological activities of striatum medium spiny neurons (MSN) neurons in slices and used behavior metrics to evaluate the effects of psychostimulants such as cocaine on the WT and MU.^[^ ^8^, ^9^ ^]^ However, these studies either only indirectly reflect extracellular DA levels in the striatum or offer snapshots of the DA transmission dynamic. To better characterize the interactions between the *Clock* gene and the DA system, a tool that can directly measure extracellular tonic DA concentration in conjunction with electrophysiological activities over an extended period in the striatum will greatly help to elucidate the mechanisms underlying *Clock*’s regulation of the DA system.

Implantable microelectrode arrays (MEA) with metal sites have been extensively utilized as a state‐of‐the‐art technique to record electrophysiological activities in the brain.^[^ ^18^, ^19^, ^20^, ^21^, ^22^, ^23^, ^24^ ^]^ On top of the electrical recording capability, MEAs can also be used as a versatile platform for incorporating additional functionalities (electrochemical sensing, light or drug delivery) and have achieved a considerable level of success for acute applications.^[^ ^19^, ^20^, ^22^, ^24^, ^25^, ^26^, ^27^, ^28^, ^29^, ^30^, ^31^, ^32^ ^]^ The idea of using MEAs for in vivo electrochemical DA sensing and electrophysiology recording has been shown to be possible under acute conditions.^[^ ^28^, ^29^, ^33^ ^]^ However, conventional MEAs have metal sites that have low specificity and sensitivity toward DA. Carbon has been the go‐to material for the electrochemical detection of DA due to its chemical inertness and high sensitivity toward DA.^[^ ^2^, ^34^ ^]^ There are generally two approaches to make MEAs with carbon sites: assembling carbon fiber arrays (CFA) or lithographically fabricating MEAs with glassy carbon (GC) sites through a pyrolysis process.^[^ ^24^, ^26^ ^]^ Unfortunately, CFA fabrication process requires manual assembly that prevents large‐scale batch fabrication of the devices,^[^ ^24^, ^26^ ^]^ while the high‐temperature pyrolysis process of GC MEAs increases fabrication complexity and limits substrate choices. Furthermore, although both carbon fiber and GC microelectrodes are excellent for Fast Scan Cyclic Voltammetry (FSCV) detection of phasic DA, their low surface area characteristics result in high impedances that are undesirable for tonic DA detection and electrical recording and stimulation.^[^ ^35^ ^]^ Previous works using carbon‐based electrodes for in vivo applications have been limited to phasic DA detection only.^[^ ^2^, ^24^, ^26^, ^35^, ^36^ ^]^ Alternatively, depositing organic conducting polymer coating on metal sites of conventional MEAs has been well known for drastically reducing electrode impedance and improving the recording and stimulation functionality of metal MEAs.^[^ ^37^, ^38^, ^39^, ^40^ ^]^ Organic conducting polymer coatings such as poly(3,4‐ethylenedioxythiophene) (PEDOT) doped with carbon material such as graphene oxide and carbon nanofibers have been shown to be excellent materials for electrochemical detection of neurochemicals.^[^ ^41^, ^42^, ^43^ ^]^ Particularly, PEDOT doped with acid‐functionalized carbon nanotubes (PEDOT/CNT) have been successfully utilized for chronic electrophysiology recording and stimulation with stiff silicon‐based MEAs.^[^ ^19^, ^44^ ^]^ Using an optimized Square Wave Voltammetry (SWV) waveform, PEDOT/CNT coatings also enable tonic DA detection with great selectivity, sensitivity, and high temporal resolution.^[^ ^36^, ^37^, ^40^ ^]^

Lastly, there's overwhelming evidence in the literature showing that the inflammatory response to electrode implants may compromise MEAs’ chronic performance.^[^ ^45^, ^46^ ^]^ The mechanical mismatch between traditional stiff MEAs and soft brain tissue has been shown to play an important role in chronic inflammation.^[^ ^47^, ^48^ ^]^ Minimizing the mechanical mismatch by using flexible MEAs can alleviate adverse tissue response.^[^ ^21^, ^49^, ^50^, ^51^ ^]^ Thus, to achieve chronic sensing and recording, we opted to build the dual‐function MEAs on flexible substrates.

**Figure 1:**
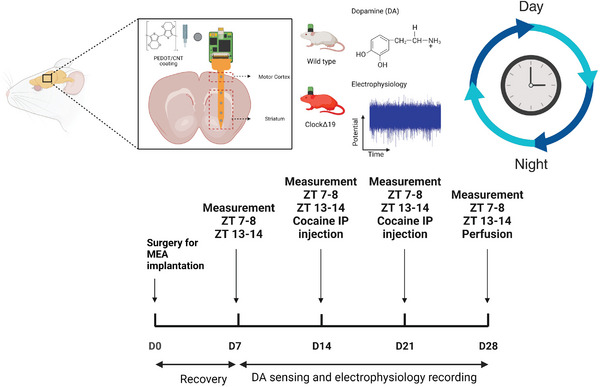
Summary of the experimental design and timeline. Custom‐fabricated flexible MEAs with PEDOT/CNT coatings on all channels were implanted 4.5 mm deep into the striatum of the mouse brain. Both extracellular tonic DA dynamics and electrophysiology were measured at ZT 7‐8 and ZT 13‐14 weekly for 4 weeks in WT and MU mice.

In the current work, PEDOT/CNT coating was deposited on microfabricated flexible 20‐channel polyimide MEAs and implanted in both WT and MU mice for 4‐weeks. 6 of the 20 microelectrode sites were located in the cortex and the remaining 14 were in the striatum (**Figure** **1**). Electrochemical Impedance Spectroscopy (EIS) measurement, SWV DA sensing, and electrophysiology recording were performed at ZT 7–8 and ZT 13–14 time points weekly. On days 14 and 21, cocaine injections were given to validate the DA signals. Animals were perfused at the end of 4 weeks and brains were sliced for immunohistology study. To the best of our knowledge, this is the first demonstration of chronically implanted dual‐function flexible MEAs that can stably detect tonic DA and record electrophysiology in both striatum and cortex regions of the mouse brain. This is also the first study that tracks and compares tonic DA dynamic and electrophysiological activities between WT versus MU at different times of the day and reveals *Clock*’s involvement in DA regulation.

## 2 Experimental Section

### 2.1 Materials

3,4‐ethylenedioxythiophene (EDOT), cocaine hydrochloride C‐II, nitric acid (95% fuming), and sulfuric acid were purchased from Sigma‐Aldrich (St. Louis, MO, USA). Muti‐wall carbon nanotubes (CNT) (10–20 nm diameter, 10–30 µm length, 95%, 200–350 m^2^ g^−1^) were purchased from a cheap tube (Grafton, VT, USA).

### 2.2 MEA Fabrication

The flexible MEAs were fabricated using an optimized procedure from previous work.^[^ ^21^ ^]^ A 4‐inch Si wafer with a 500 nm thick SiO_2_ layer (University Wafer Inc., USA) was first cleaned by sonicating in acetone, isopropanol, and DI water sequentially for 5 min, respectively. 1). The cleaned wafer was dried on a hot plate at 95 °C for 3 min, then the surface was cleaned and activated by O_2_ plasma using a reactive ion etcher (RIE, Trion Phantom III LT, Clearwater, FL, USA) for 90s at 200mTorr pressure and 150Watts power. The wafer was then spin‐coated with polyimide (PI) HD4100 (HD MicroSystems L.L.C. NJ, USA) at 3000 rpm for 1 min and soft baked at 95 °C for 1 min to evaporate solvent, then the wafer was exposed with a customized mask using a Quintel Q4000 mask aligner (NEUTRONIX QUINTEL, CA, USA) at a dose of 400mJ cm^−2^. After exposure, the PI layer was post‐baked at 95 °C for 2 min, developed using PA‐401D developer (HD MicroSystems L.L.C. NJ, USA) for 1 min. The wafer was then rinsed and cleaned with PA‐400R (HD MicroSystems L.L.C. NJ, USA) and dried and baked at 95 °C for 1 min. The wafer was cured at 200 °C for 30 min and 350 °C for 1 h in an OTF 1200 Serie tube furnace purged with N_2_ (MTI Corporation, CA, USA). 2). The wafer was then treated with O2 plasma to clean, activate, and roughen the PI with RIE for 60 s at a pressure of 200mTorr and 150 W power. The treated wafer was then spin‐coated with AZ P4210 photoresist (MicroChemicals, Germany) at 4000 rpm for 1 min and baked at 105 °C for 2 min for resist curing. After baking, the wafer was exposed using MLA with a dose of 250mJ cm^−2^, then developed using AZ400k 1:4 developer (MicroChemicals, Germany), cleaned by water rinse, and dried with N2 gas flow. A mild 120 s RIE O2 plasma treatment at pressure 600 mTorr and 60 W power was performed to clean the surface before metal deposition. A 15 nm Ti adhesion layer, 100 nm Au, and 20 nm Pt layer were evaporated on the wafer using an Electron Beam Evaporator Plassys MEB550S (Marolles‐en‐Hurepoix, France). The metal was then lifted off in acetone overnight. 3). The next day, the wafer was first rinsed with water, dried under N_2_ flow, and cleaned by O2 plasma for 60 s at 200 mTorr and 150 W, then spin coated with HD4100 for the middle PI insulation layer at 5000 rpm for 1 min and soft baked at 95 °C for 5 min. The wafer was then flood exposed using mask aligner with a dose of 400 mJ cm^−2^, post‐backed, and cured at 200 °C for 30 min and 350 °C for 1 h in tube furnace purged with N_2_. 4). Then step‐2 was repeated to deposit another layer of metal traces and sites. 5). The wafer was spin coated with HD4100 for the last PI insulation layer at 3000 rpm for 1 min and soft baked at 95 °C for 5 min. The wafer was then flood exposed using mask aligner with a dose of 400 mJ cm^−2^, post‐backed, and cured at 200 °C for 30 min and 350 °C for 1 h in tube furnace purged with N_2_. 6). The wafer was then spin‐coated with AZ P4620 photoresist at 2000 rpm for 1 min and baked at 105 °C for 10 min for resist curing. After baking, the wafer was exposed using MLA with a dose of 950mJ cm^−2^, then developed using AZ400k 1:4 developer, cleaned by water rinse, and dried with N2 gas flow. The sites and contact pads are dry etched open using RIE (200mTorr, 180 W, 180s, 50sccm O_2_, 2sccm SF_4_. 180s etching with 120s cool down each cycle. 5 cycles). 7). The flexible PI MEAs were released from the wafer using buffered oxide etchant (1:7) (MicroChemicals, Germany) in an acid hood for 8hr to etch away the SiO_2_ layer. Customized PCBs (Wavemed, Italy) were mounted with one 23‐contact ZIF (Digikey, USA) and two 16‐channel Omnetics connectors (Omnetics connector corporation, MN, USA). ZIF connectors were used to interface with flexible MEAs, and Omnetics connectors were used to interface with the potentiostat and electrophysiology recording system.

### 2.3 Electrochemistry Methods

All electrochemical procedures were conducted using a three‐electrode design (working electrode: individual MEA electrodes; reference electrode: Ag/AgCl; counter electrode: Pt (in vitro)/stainless‐steel bone screw (in vivo). An Autolab potentiostat/galvanostat, PGSTAT128N (Metrohm, Herisau, Switzerland) was used for all square wave voltammetry (SWV) procedures and electrochemical Impedance spectroscopy (EIS) measurements (in vivo and in vitro). SWV potential was swept from scanned −0.2 to 0.3 V using a 25 Hz pulse frequency, 50 mV pulse amplitude, and a 5 mV step height. A liner scan from 0.3 to 0 V at 1 v s^−^1 was applied after SWV waveform. Potential was held at 0 V between scans.

#### 2.3.1 In Vitro Calibration, Selectivity, and Fouling Test

In vitro DA calibrations were performed using freshly prepared DA standard solutions dissolved in 1x PBS with or without interfering agents. SWV selectivity for DA at PEDOT/CNT‐coated MEAs was determined by performing SWV measurements in a cocktail solution containing AA (200 µM), DOPAC (20 µM), UA (20 µM), and serotonin (100 nm). Selectivity was assessed via direct comparison of DA sensitivity in the presence and absence of interfering agents.

For fouling test, EIS measurements were first performed on freshly coated MEAs, then MEAs were soaked in PBS solution containing 10 mg ml^−1^ of bovine albumin for 1 h at room temperature. MEAs were then rinsed with 1x PBS before taking EIS measurements again.

#### 2.3.2 CNT Functionalization and PEDOT/CNT Deposition

CNTs were functionalized following previous methods.^[^ ^19^, ^22^ ^]^ In brief, 200 mg of multiwalled carbon nanotubes into 25 ml of concentrated nitric acid and 75 ml of concentrated sulfuric acid. This solution was sonicated for 2 h, and then stirred overnight at 35 °C. The solution was dialyzed in a DI water bath until the solution became pH‐neutral. The water bath was changed every 12 h. Samples were vacuum dried and stored at 4 °C.

For PEDOT/CNT deposition, 1 mg mL^−1^ of functionalized CNTs was resuspended in DI H2O by sonication for 10 min. EDOT was added to this solution to a concentration of 0.01 m. The solution was then sonicated for 10 min using a Q500 probe sonicator (Qsonica L.L.C, Newtown, CT, USA). The electrochemical deposition was performed using chronocoulometry. The applied voltage was 0.9 V, with a charge cut off at 150 mC cm^−2^.

### 2.4 Scanning Electron Microscope (SEM) Characterization

SEM images were obtained with an FEI Scios Dual Beam System (ThermoFisher Scientific, Waltham, MA, USA) with a 5 kV, 0.1 nA beam and a 7 mm working distance.

### 2.5 Animal Housing and Breeding

Mice were group housed on a 12/12 light/dark cycle (lights on 7 a.m., lights off 7 p.m.) with food and water ad libitum. *Clock*Δ19 mice on a Balb/c mixed background were bred as heterozygotes to produce WT and homozygous MU littermates. Female *Clock* mutant (*Clk*Δ19/*Clk*Δ19) and wild‐type (+/+) littermate controls, 15–19 weeks old, were used in all studies. Mice were originally from Dr. Joseph Takahashi.

### 2.6 Surgical Procedure

All animal work was performed under the guidelines of the University of Pittsburgh Institutional Animal Care and Use Committee (IACUC). The approved protocol ID is 22109970. Mice were anesthetized under isoflurane (2.5%) and head‐fixed in a stereotaxic frame (David Kopf Instruments, Tujunga, CA, USA). Animal body temperature was maintained at 37 °C using an isothermal pad connected to a SomnoSuite system (Kent Scientific Corporation, Torrington, CT, USA). Heart rates were monitored using SomnoSuite system as well. A holder was used to secure the custom‐designed PCBs. TDT recording system (RX5, 16‐channel Medusa amplifier, Tucker Davis Technologies (TDT), Alachua, FL) was used to record electrophysiology data. Three skull screws were carefully positioned above the left striatum, right visual cortex, and left visual cortex of the mice as illustrated in Figure 4a). A 0.7 mm diameter window above the ventral striatum of the right hemisphere was opened using a motorized drill. The coordinates for the center of the window were 1 mm posterior to Bregma and 1.1 mm lateral to the midline. The flexible shank was temporarily glued to a 50 µm diameter Tungsten wire shuttle with 30% polyethylene glycol (PEG, MW 20 kDa). The PCB/MEA assembly, tungsten wires, and PEG solution were UV sterilized for 10 min before surgery, and tungsten wires were attached to MEA shanks using sterilized PEG before implantation. The fully assembled device was inserted into the brain using a motorized micromanipulator from NeuralGlider (Actuated Medicine, Inc, Bellefonte, PA, USA) 4.5 mm deep into the brain. A Pt counter wire was connected to the skull screw on the contralateral hemisphere for electrophysiology recording. Another Pt wire was connected to the screw above the contralateral visual cortex. Both wires were soldered to corresponding ground wires on the PCB.

### 2.7 In Vivo DA Sensing and Electrophysiology Recording

Animals to first anesthetized with 2% ISO, then the head‐stage for either potentiostat or recording system was connector to the animal head through and imbedded PCB using omnetics connectors. SWV DA was applied for 10 min each session. A fresh Ag/AgCl was used for each session and inserted subcutaneously. Electrophysiology recording was collected 5 min long. A 20 min SWV DA detection was performed after cocaine injection (10 mg k^−1^g). Cocaine was dissolved in PBS and delivered through Intraperitoneal injection. The neural signal from recording microelectrodes was amplified using a 16‐channel Medusa preamplifier and recorded with an RX5 processor at 25 kHz.

### 2.8 Immunohistochemistry

According to the University of Pittsburgh IACUC approved methods, mice were scarified at the end (4 weeks). Each animal was deeply anesthetized using 80–100 mg k^−1^g ketamine, 5–10 mg k^−1^g xylazine cocktail. Once mice were unresponsive to tail/toe pinches, animals were transcardially perfused using 1 X phosphate buffered saline (PBS) flush at <100 mmHg followed by 4% paraformaldehyde (PFA) at <100 mmHg. Mice were decapitated and the skulls were removed to post‐fix the brain in a 4% PFA at room temperature for 12 h. Then, brains were soaked in a 15% sucrose (Sigma–Aldrich Corp., St. Louis, Missouri) bath at 4 °C overnight followed by a 30% sucrose solution for 24 h. Brains were then carefully frozen in a 2:1 20% sucrose in PBS:optimal cutting temperature compound (Tissue—Plus O.C.T. Compound, Fisher HealthCare, Houston, TX) blocking media blend with dry ice. Frozen tissue was then horizontally sectioned into 25 µm thick sections along the tract of the probes using a cryostat (Leica CM1950, Buffalo Grove, IL).

Cortical sections of implanted and non‐implanted hemispheres were mounted on the same slide for comparison and staining for each antibody combination was performed at the same time to minimize variability. Antibodies to visualize astrocytes (GFAP, 1:500, Z033401 Dako), macrophage/microglia IBA‐1(microglia, 1:500, NC9288364 Fisher).

Tissue sections were rehydrated in 1 x PBS for 2×5 min. The tissue was then incubated in 0.01 m sodium citrate buffer for 30 min at 60 °C. Then, a peroxidase block (PBS with 10% v/v methanol and 3% v/v hydrogen peroxide) was performed for 20 min at room temperature (RT) on a table shaker. Next, tissue sections were incubated in carrier solution (1 X PBS, 5% normal goat serum, 0.1% Triton X‐100) for 30 min at RT. Lastly, the tissue sections were blocked with Alexa Flour 647‐conjugated AffiniPure Fab Fragment goat anti‐mouse IgG (IgG, 1:16, 115‐607‐003 Jackson ImmunoResearch Laboratories, Inc.) or Fab fragment only (1:13, 115‐007‐003, Jackson ImmunoResearch Laboratories, Inc.) for 2 h then rinsed 6 times each 4 min. Following blocking, sections were incubated in a primary antibody solution consisting of carrier solution and antibodies listed above overnight (12–18 h) at RT. Sections were then washed with 1 x PBS for 3×5 min and incubated in a carrier solution and secondary antibodies (1:500, Alexa Flour 488 goat‐anti mouse, Invitrogen, and 1:500 Alexa Flour 568 goat‐anti rabbit, Invitrogen, 1:500 Alexa Flour 633 goat‐anti chicken, Invitrogen) for 2 h at RT. Then sections were rinsed with PBS for 3×5 min and exposed to Hoechst (1:1000, 33342 Invitrogen) for 10 min and washed in PBS for 3×5 min, then covered by coverslip with Fluoromount‐G (Southern Biotech, Associate Birmingham, AL).

### 2.9 Data Analysis

Each SWV response was first filtered using a zero‐phase, forward, and reverse (using the filtfilt function on MATLAB), low‐pass, third‐order Butterworth digital filter with the 3 dB cutoff at a normalized frequency of 0.2 (2 Hz). The fit for the linear baseline was determined using a two‐step peak extraction method consisting of an iterative peak localization algorithm. First, a linear baseline was initialized with two signal points on either side of a user‐selected peak maximum voltage (≈0.18 V). Signal points used to construct the baseline were iteratively updated to produce a final baseline that maximized the subtracted peak amplitude. The resulting linear fit intersected boundary points at either side of the DA redox peak profile. The five data points immediately adjacent to the upper and lower bounds were then modeled using linear fitting and subtracted from the raw SWV response for the purpose of peak extraction as demonstrated in Figure S1 (Supporting Information). All extracted peak current was converted to DA concentration using the table and equation shown in Figure S1 (Supporting Information).

Raw neural recording data was filtered between 300 and 10 k Hz. Threshold crossing events were identified by using a fixed negative threshold value of 3.5 standard deviations. Plexon offline sorter (Plexon Inc Dallas, TX, USA) was used to identify single units. A 3D PCA feature space was used to identify waveform features and K‐means clustering method was used to identify individual units. K‐means was set up using an adaptive standard EM between 2 and 5. The Signal‐to‐Noise (SNR) ratio of sorted units was calculated and units with an SNR above 4 were included in the analysis. The SNR is calculated using the Equation (1) below:

(1) $$SNR=\frac{V_{p2p}}{\sigma \left(V_{noise}\right)}$$

where V_p2p_ is peak to peak amplitude of single units, and σ(V_noise_) is the standard deviation of the high pass filtered stream.

The single unit yield (SUY) is calculated using the Equation (2) below:

(2) $$SUY=\frac{N_{active}}{N_{total}}\;*100\%$$

where N_active_ is number of electrode sites that recorded single units, N_total_ is the total number of functional electrode sites.

A customized MATLAB script was used to calculate SNR, SUY, spike rates, and p2p amplitudes. Statistical analysis and results plotting were done using GraphPad Prism 10.0.0 and MATLAB 2019a. Data described in the text are mean ± SM unless specified otherwise. Schemes were drawn with BioRender.

## 3 Results and Discussion

### 3.1 MEA Fabrication and In Vitro Stability of PEDOT/CNT Coating

**Figure 2:**
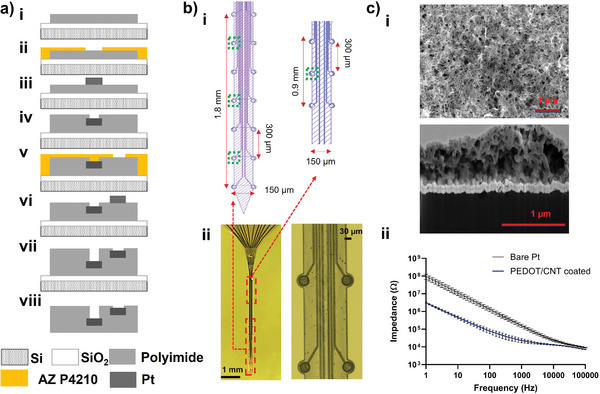
Schematic demonstration of the fabrication process, MEA layout, and PEDOT/CNT coating characterization. a). Step‐by‐step fabrication process of the customized 20 channel PI MEAs. i. A polyimide insulation layer (≈5 µm thick) spin‐coated on a SiO_2_ wafer and patterned with photolithography. ii. Positive photoresist AZP4210 spin‐coated and patterned with photolithography. iii. 15 nm Ti adhesion layer, 100 nm Au, and 20 nm Pt layer evaporated on substrate and lift‐off in acetone. iv. Middle insulation layer polyimide spin‐coated and photolithography patterned. v‐vi. Repeat ii‐iii for metal layer deposition and patterning. vii. The last insulation layer polyimide spin‐coated and patterned. viii. MEAs are released using HF acid. b). MEA design layout, optical images of the fabricated MEAs. i. Shank is 150 µm wide, 12 µm thick, and 5 mm long, with 300 µm spacing between individual sites. There are 20 channels in total, 4 sites in green boxes were only used for DA sensing. ii. Optical image of the MEA shank. Region 1 (striatum) has 14 sites and region 2 (cortex) has 6 sites. In set shows PEDOT/CNT coating pt sites. The sites are 35 µm in diameter. c). i. SEM image of PEDOT/CNT coating on top of Pt sites and cross‐section view of the rough and porous coating. Coating is ≈ 0.5 µm thick. ii. EIS of MEAs before and after depositing PEDOT/CNT coatings has small variations. (14 MEAs, n= 255 sites).

Using photolithography microfabrication, we batch‐produced flexible MEAs with high channel counts and customized channel mapping design. We used a multilayer approach to increase channel count while maintaining a small footprint (**Figure** **2**a). The MEA has 20 electrode sites along the 4.5 mm long shank. 14 out of 20 sites at the lower shank region spanning 1.8 mm are designed to cover the entire depth of the mouse striatum, while the top 6 sites spanning 0.9 mm survey the motor cortex (Figure 2b). PEDOT/CNT coatings were electropolymerized on all 20 sites and dry‐stored before surgery. The electropolymerization method enables precise control of coating thickness by controlling the deposition charge density, and the resulting film exhibits a nanofibrous and porous morphology with ≈0.5 µm thickness (Figure 2c,i). The fabrication and coating deposition processes were highly reproducible as reflected by the minimum variation in the EIS measurement of all 14 devices used in the experiments (Figure 2c, ii). In our previous studies, PEDOT/CNT coatings on carbon fiber electrodes have shown excellent selectivity over a range of neurochemical interferences, including 3,4‐dihydroxyphenyl‐acetic acid (DOPAC) and ascorbic acid (AA), both of which have redox potentials similar to that of DA.^[^ ^37^ ^]^ In this work, we confirmed the selectivity of PEDOT/CNT coated on flexible MEAs by showing unaffected SWV current response to DA in the presence of interfering compounds such as uric acid (UA, 20 µm), AA (200 µm), 5‐hydroxytryptamine serotonin (100 nm), and DOPAC (20 µm) (Figure S2, Supporting Information). PEDOT/CNT coatings contain negatively charged acid‐functionalized CNTs.^[^ ^37^, ^52^ ^]^ DOPAC and AA also carry negative charges at physiological pH (≈7.4). Electrostatic repulsion prevents negatively charged interfering molecules from approaching the sensor surface and contributing to the SWV redox currents. This phenomenon is similar to Nafion, a negatively charged coating typically deposited onto the surface of CFEs to provide electrostatic separation of positively charged neurochemicals over negatively charged interferences, such as AA and DOPAC.^[^ ^53^ ^]^

**Figure 3:**
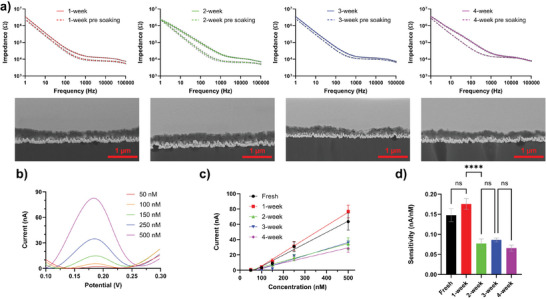
In vitro stability test of PEDOT/CNT coating. a). Impedance of coatings before and after soaking in PBS of each time point and cross‐section SEM images of PEDOT/CNT after soaking. b). SWV waveform for DA calibration. c). Calibration curve comparison of freshly coated MEAs vs MEAs soaked in PBS for up to 4 weeks. d). Quantified sensitivity comparisons of fresh coating and coating soaked in PBS (n=12, n=15, n=16, n=20, n=17, for fresh, 1‐week, 2‐week, 3‐week, and 4‐week conditions, respectively. One‐way ANOVA with Šídák's multiple comparisons test. ^****^ *p*<0.0001.).

The stability of PEDOT/CNT coatings was also tested in vitro. PEDOT/CNT‐coated MEAs were soaked in Phosphate‐buffered saline (PBS) at 37 C for up to 4 weeks. Each week one MEA was retrieved from PBS bath for EIS measurement and DA sensing calibration. EIS spectra were relatively stable throughout the 4 weeks of soaking, with individual array before and after soaking showing a small impedance increase over a wide frequency range (**Figure** **3**a). The cross‐section of the coating on MEAs from all time points showed no changes in coating thickness and morphology and no signs of mechanical failure (Figure 3a), indicating excellent coating stability. MEAs were exposed to a series of DA solutions with known concentrations (50nM‐500 nm) while performing SWV for sensor calibration (Figure 3b). The size of DA redox peak ≈ 0.18 V was extracted and plotted against DA concentration to acquire calibration curves (Figure S1, Supporting Information). The calibration curves of DA sensing showed a linear response toward a gradient of DA concentrations at all time points. (Figure 3c). We defined the sensitivity by the slope of the calibration. A drop in sensitivity was observed at the 2‐week time point, but remains high and stable thereafter (Figure 3d). The increase in impedance after the soaking test and the reduction of sensitivity at week 2 could be the result of loosely bound CNTs leaching from the PEDOT/CNT polymer.^[^ ^46^, ^47^ ^]^ We found that the 25 Hz (scanning frequency of SWV) impedance negatively correlates with the sensitivity (Figure S3b, Supporting Information) and may be used as a parameter to predict sensors’ performance. The limit of detection (LOD) of the PEDOT/CNT‐coated flexible MEAs calculated as 3 x standard deviation of the noise of SWV blank conditions, over 4 weeks in vitro is shown in Figure S3a (Supporting Information).

### 3.2 In Vivo Stability of PEDOT/CNT Coatings and MEAs

**Figure 4:**
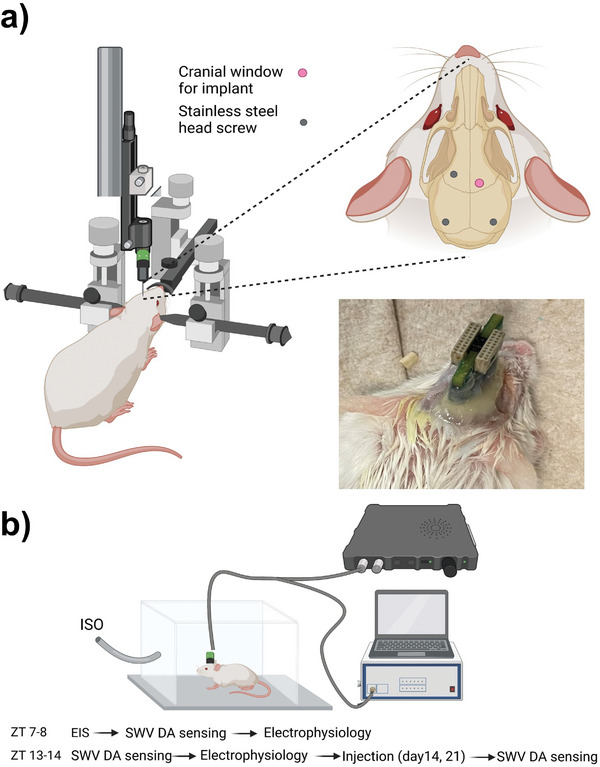
Surgical set‐up and experiment session design. a). Mice were anesthetized with isofluorane and placed under a stereotaxic frame. A motorized micromanipulator was used to hold and implant the MEA.)). Three head screws were used as anchoring points for dental cement fixture, and two of them on the contralateral side of the implant were used as ground for electrophysiology and electrochemistry measurement respectively. A customized PBS with 2 omnetics connectors was used to connect MEAs with the recording and potentiostat system, respectively. b). For each experiment session, mice were lightly anesthetized for SWV, and electrophysiology recording at ZT 7‐8 and ZT 13‐14. Cocaine injection was given after ZT 13‐14 baseline measurement, after which SWV DA sensing was performed again to measure effects of cocaine injection for day 14 and day 21.

For in vivo surgery, MEAs were positioned using stereotaxis and implanted 4.5 mm deep into the striatum of mouse brains using a motorized holder (**Figure** **4**a). Three head screws were used as anchoring points for dental cement fixture, and two of them were placed on the contralateral side of the implant and served as ground for electrophysiology and electrochemistry measurement respectively. A freshly prepared Ag/AgCl wire was inserted subcutaneously into the back of the mice and used as reference for all electrochemistry measurements and removed afterward. We have previously found this method provides a more reliable reference electrode for in vivo measurement than permanently implanted Ag/AgCl.^[^ ^54^ ^]^ Customized PCBs with 2 separate omnetics connectors were used to connect MEAs with the TDT recording and Autolab potentiostat respectively. EIS was measured at day 0 after surgery, and once per week at ZT 7. Electrophysiology recording was performed once at ZT 13–14 on day 0, and then twice between ZT 7–8 and ZT 13–14 on d7, 14, 21, and 28. DA sensing was performed once per week starting on day 7 and twice per day immediately before each electrophysiology recording session. For DA sensing, the first week SWV measurement was very noisy, possibly due to the unstable interface after surgery where undissolved PEG residue, tissue debris, and blood accumulate and resolve around the shank.^[^ ^46^ ^]^ Mice were lightly anesthetized during each session (Figure 4b).

**Figure 5:**
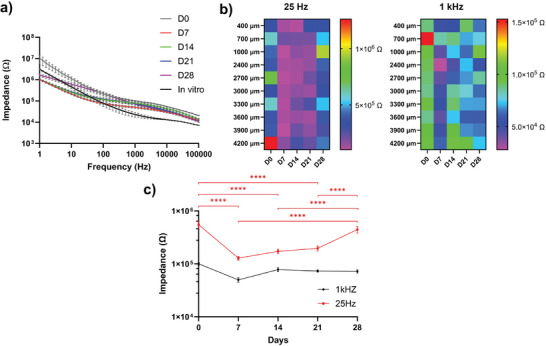
Impedance of MEAs used in vivo over the 4‐week period. a). Averaged impedance spectroscopy changes over 4 weeks. The initial 2 weeks showed some variations in impedance that stabilized at week 2. b). Impedance of 25 Hz and 1 kHz frequency band. 25 Hz is the scanning frequency of SWV. Impedance was mostly stable between day 7 and day 21. Some variations were observed on day 0 and day 28. A spatial pattern can be observed for both time points. c). Average impedance across depth for 25 Hz and 1 kHz. Day 7, 14, and 21 showed significantly lower 25 Hz impedance than day 0 and day 28. No statistical difference was observed for all time points for 1 kHz impedance. (10 mice, 5 MU and 5 WT combined. Two‐way ANOVA with Tukey's multiple comparisons test. **** p<0.0001.)

Although previous work has demonstrated stable chronic recording performance of PEDOT/CNT coatings on stiff Si probes, stability and chronic performance of PEDOT/CNT coatings on flexible MEAs has yet to be investigated.^[^ ^19^ ^]^ Flexible MEAs can flex with the brain tissue and reduce mechanical stress on the tissue; However, such flex may present a mechanical challenge to the PEDOT/CNT coatings on top of the sites.^[^ ^55^ ^]^ Additionally, the continuous scanning of SWV for DA detections adds another level of electrochemical stress to the PEDOT/CNT coating.^[^ ^44^ ^]^ Therefore, it is important to evaluate the stability of the PEDOT/CNT coating on flexible MEAs. Impedance measurements were used to gauge the stability of the coating and MEAs during the 4‐week period. We observed that the EIS of all in vivo MEAs were relatively stable except for variations on early time points (**Figure** **5**a). Day 0 has higher impedances at all frequencies than in vitro impedance, and this may be attributed to undissolved PEG residue, tissue debris, blood plasma fouling, and microglia processes attachment.^[^ ^56^ ^]^ The impedance decreased at day 7 when tissue healing and clearance of debris and blood were mostly complete.^[^ ^57^ ^]^ The EIS spectra shape remained mostly the same until day 21. By day 28, an increase in impedance magnitude especially at the low frequency range is observed which might be the result of glia sheath consolidation.^[^ ^46^, ^58^ ^]^ The impedance modulus at 25 Hz and 1 kHz were specifically analyzed, because 25 Hz is the scanning frequency of SWV, and 1 kHz is the neuronal firing frequency relevant to single unit recording. The spatial map of 25 Hz impedance over depth showed that impedance was mostly stable between day 7 and day 21 with little variation among depths. Both day 0 and day 28 had higher impedance with higher variation across depth (Figure 5b). On the other hand, 1 kHz impedance was mostly stable, and spatially distinct patterns can be observed for all time points besides day 0. By averaging across all tissue depths, the 25 Hz and 1 kHz impedance were compared over time (Figure 5c). The 25 Hz impedance was higher on d0 and d28 than d7‐21, with the overall average (across time) being 235.78 kΩ with a standard deviation of 162.44 kΩ. The 1 kHz impedance was low (74.79 kΩ ± 23.01 kΩ, mean ± SD) and showed no significant difference over time (Figure 5c). The stable and low impedance indicates good mechanical and electrical stability of the PEDOT/CNT coatings on flexible MEAs and supports high quality recording.

### 3.3 Chronic DA Dynamics in WT and MU

**Figure 6:**
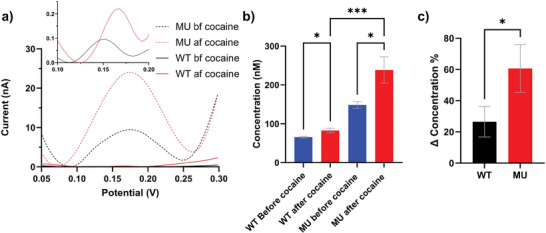
Representative SWV waveforms and effects of cocaine injections in WT and MU. a). Both WT and MU showed an increase in DA redox peak current size ≈ 0.18 V after receiving cocaine injections. Inset shows a magnified SWV waveform of WT. b). Both WT and MU had significantly increased DA concentration after cocaine injection. MU showed significantly higher DA concentration after cocaine injections compared to WT. (5 mice each group. n=24 WT before, n=14 WT after, n=55, MU before, n = 29 MU after. (One‐way ANOVA with Dunnett's multiple comparisons test, ^***^ *p*<0.0005, ^*^ *p*<0.05.) c). Normalized percent DA response induced by cocaine injection was significantly higher in MU than in WT. (5 mice each group, n=14 WT, n=29 MU. Welch's *t*‐test one‐tail, ^*^ *p*<0.05.)

The detailed process for quantifying SWV peak current and converting raw current peak to DA concentration using the established calibration curves is shown in Figure S1 (Supporting Information). SWV waveforms in the cortical regions do not have any resolved DA redox peaks at ∼0.18 V, which is consistent with the known low presence of DA in this region (Figure S4, Supporting Information). The lack of DA signals in the cortex provides a validation of our DA measurement. Next, using the cocaine injection experiments, we further validated the tonic extracellular DA detection. Cocaine is a potent psychostimulant that causes DA buildup by binding DA transporters and blocking DA reuptake.^[^ ^59^, ^60^, ^61^ ^]^ Representative SWV waveforms of the WT and MU before and after cocaine are shown in **Figure** **6**a. Both the MU and WT had an elevated DA peak current upon cocaine injections as expected, further validating that the redox peak observed in SWV indeed was DA. MU mice had a much larger current (≈ 0.18 V) compared to WT both before and after cocaine injection (Figure 6a), which corresponded to higher average DA concentration (across depth and time) (Figure 6b). This finding is consistent with previous research that showed MU had higher dopaminergic transmission activities in the VTA region in terms of both TH expression and neuron firing rate and higher DA contents with HPLC measurements of homogenized striatal tissue.^[^ ^8^, ^9^, ^17^, ^62^ ^]^ Notably, the % changes of DA concentration induced by cocaine injection were also significantly higher in MU than WT (Figure 6c).

**Figure 7:**
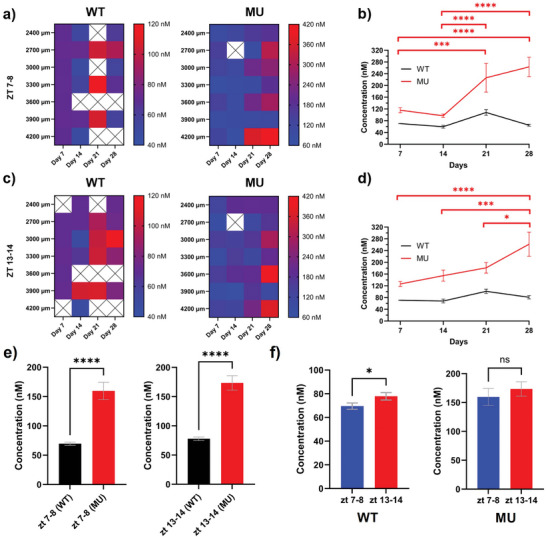
Spatial and temporal DA concentration dynamics in WT and MU. a). DA concentration along the depth of striatum at ZT 7‐8 over 4‐week period. Blank boxes with a X indicate channels that did not resolve a DA peak. b). Average DA concentration across tissue depth over time. DA concentration of WT was mostly stable over time. MU had significantly higher DA concentration on day 21 and day 28, compared to day 7 and day 14. (5 mice each group, Two‐way ANOVA with Tukey's multiple comparisons test. ^****^ *p*<0.0001, ^***^ *p*<0.0005.) c). DA concentration profile along the depth of striatum at ZT 13‐14 over 4‐week period from both WT and MU. d). Average DA concentration across tissue depth over time. DA concentration of WT was again stable over time. MU had a significantly higher DA concentration on day 28, compared to day 7, day 14, and day 21. (5 mice each group, Two‐way ANOVA with Tukey's multiple comparisons test. ^****^ *p*<0.0001, ^***^ *p*<0.0005, ^*^ *p*<0.05.) e). MU had significantly higher DA concentration at ZT 7‐8 and ZT 13‐14 compared to WT. (5 mice each group, WT, n=43 (ZT 7‐8), n = 42 (ZT 13‐14). MU, n=65 (ZT 7‐8), n = 66 (ZT 13‐14), Welch's t test, ^****^ *p*<0.0001.) f). Average DA concentration across time and depth in WT showed significantly higher DA concentration at ZT 13‐14 compared to ZT 7‐8. (5 mice, n=43 (ZT 7‐8), n = 42 (ZT 13‐14), Welch's *t*‐test. ^*^ *p*<0.05). MU had similar levels at both ZT 7‐8 and ZT 13‐14. (5 mice each group, n=65 (ZT 7‐8), n = 66 (ZT 13‐14), Welch's *t*‐test)

Diurnal variation of DA concentration has been reported in the literature, with minimum and half maximum DA levels showing around ZT 7–8 and ZT 13–14, respectively.^[^ ^16^ ^]^ Therefore, these time zones were chosen to have a clear contrast of tonic dopamine concentrations. The spatial distribution and temporal changes of DA concentration over the 4‐week period at ZT 7–8 and ZT 13–14 were investigated in both the WT and MU (**Figure** **7**). At ZT 7–8, both WT and MU showed distinct spatial heterogenous DA concentrations along the depth of the striatum (Figure 7a). By averaging DA levels across the tissue depth, different temporal patterns of DA concentrations were observed. MU had a significantly higher DA concentration on day 21 and day 28, compared to day 7 and day 14 (Figure 7b). WT showed stable DA dynamics with a slight increase on day 21, though not statistically significant. Similarly, at ZT 13–14 both WT and MU showed spatial patterns of DA distribution across different time points and tissue depths (Figure 7c). When the DA concentration was averaged across tissue depth and compared over time, WT again showed a mostly stable DA response over 4‐weeks (Figure 7d). However, MU had a significantly higher DA concentration on day 28 than d7, d14 and d21. Previous studies have found that *Clock* mutant mice had an increase in cocaine sensitization compared to WT.^[^ ^9^ ^]^ Sensitization (also called reverse tolerance) is a phenomenon where each intermittent cocaine dose produces a larger behavioral response than the previous dose.^[^ ^9^, ^17^ ^]^ The increase in DA levels with each cocaine dose in the MU animals is consistent with a greater behavioral locomotor response in these animals observed in previous research.^[^ ^9^ ^]^ Like the result in Figure 6, MU group has significantly higher DA concentration both at ZT 7–8 and ZT 13–14 compared to WT (Figure 7e). Next, we investigated DA dynamics at different times of the day in both WT and MU. Averaged across all time points and tissue depth, WT showed higher DA concentration at ZT 13–14 than at ZT 7–8 (Figure 7f). The diurnal changes in DA level in WT are consistent with previous research results using microdialysis.^[^ ^14^, ^16^ ^]^ Meanwhile no difference in DA concentration was observed between ZT 7–8 and ZT 13–14 for the MU. The loss of diurnal DA change in the *Clock* mutants indicates the possible role of the *Clock* gene in regulating striatal DA level, and this is consistent with the manic and hyperactive behaviors observed from previous studies as variation of DA levels is known to be directly linked with locomotor activity.^[^ ^9^, ^17^, ^63^ ^]^

Our PEDOT/CNT coated MEAs demonstrated for the first time a direct measurement of extracellular tonic DA level in the striatum of both WT and MU mice for a 4‐week period. Unique DA spatial distribution patterns were observed along the depth of the striatum and across 4 weeks with high spatial resolution (14 channels uniformly distributed over 1.8 mm). The heterogenous distribution of DA in the WT and MU with the corresponding striatum structure is also shown in Figure S5 (Supporting Information). The heterogeneity and structural and functional complexity of striatal DA circuits have been reported by a large body of studies using anatomical, FSCV, microdialysis, and electrophysiology methods.^[^ ^64^, ^65^, ^66^ ^]^ Our technique has a much higher temporal resolution (≈4s each scan) and spatial resolution compared to microdialysis—the commonly used analytical technique (minutes to hours sampling period and averaging over 0.0314 mm^3^ volume of tissue).^[^ ^67^, ^68^, ^69^ ^]^ Previous microdialysis studies reported 10–20 nm basal DA in rodent striatum,^[^ ^70^, ^71^ ^]^ which is lower than the 60–80 nm reported here in WT animals. The large footprint of microdialysis probes induces severe local tissue damage that significantly decreases extraction efficiency and results in the underestimation of analyte concentrations.^[^ ^72^, ^73^, ^74^, ^75^, ^76^ ^]^

### 3.4 Chronic Electrophysiology Recording Quality in WT and MU

**Figure 8:**
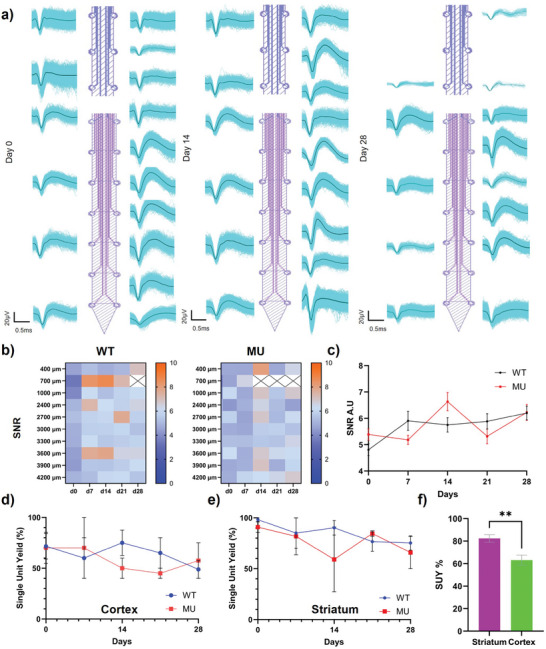
Electrophysiology recording performance of the PEDOT/CNT MEA implanted in WT and MU over the 4‐week period. a). Representative single unit waveforms on day 0, day 14, and day 28. b). Spatial mapping of SNR of WT and MU over 4 weeks. c). SNR changes over time of WT and MU. No difference was observed between WT and MU. (4 mice each group. Two‐way ANOVA with Šídák's multiple comparisons test.) d) & e). Single unit yield over time of cortex and striatum regions in WT and MU. Single unit yield was stable overall in both brain regions of WT and MU. f). Single unit yield comparison between the cortex and striatum. The striatum region had a significantly higher yield. (n=30 striatum, n=28 cortex, Welch's *t*‐test, ^**^ *p*<0.005)

The stability and quality of electrophysiology over the 4‐week period were investigated and compared between the two animal groups. Representative single unit waveforms from day 0, day 14, and day 28 showed that single units with high peak‐to‐peak (p2p) amplitudes and well‐defined waveforms can be obtained over the 4‐week period (**Figure** **8**a). On day 28, MEAs were still able to collect high quality units, though the total number of units and quality of some recorded units did decay, especially in the cortex region. WT and MU both had overall stable SNR over the 4‐week period with few hot spots emerging between day 7 and day 21 (Figure 8b). The SNR also had an inhomogeneous distribution over tissue depth and time. By averaging SNR across tissue depth, we found the SNR to be overall stable over the 4‐week period for both WT and MU groups with no difference between the two groups (Figure 8c).

**Figure 9:**
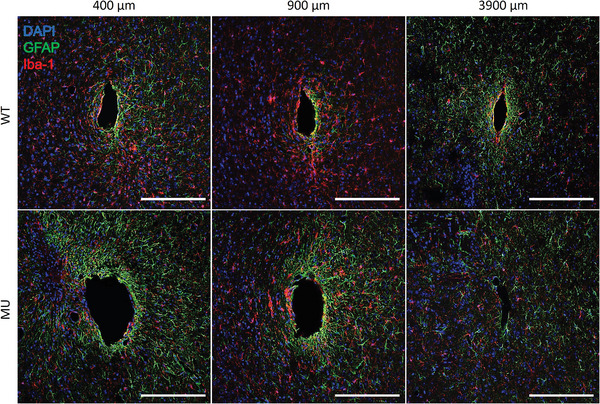
Histological investigation of inflammatory tissue response to the flexible MEA implants in WT and MU. Markers for reactive astrocytes (GFAP in red) and microglia (Iba‐1 in red) were used to investigate tissue inflammation response at different depths. Cortex region appears to have worse inflammation response and larger probe hole than the deep striatum region. Scale bar 200 µm.

Overall, SUY of both cortex and striatum region was stable and shared similar trends in WT and MU (Figure 8d,e). However, the average SUY in the cortex was significantly lower than in the striatum (Figure 8f). The results of the qualitative histology investigation also corroborate this observation. The cortex region appears to have worse inflammation responses and more extensive tissue damage than the striatum, indicated by the more intensive GFAP activities and larger size of the tissue hole (**Figure** **9**). Previous simulation studies have shown that flexible MEAs tethered to the skull experience more mechanical strain near the surface of the brain tissue than deep regions which could result in a more intensive tissue inflammation response.^[^ ^47^, ^49^, ^50^ ^]^ This could partially explain the superior SUY in the striatum. The main challenge chronic implants have to face is the foreign body responses.^[^ ^77^ ^]^ Although the acute damage from implantation surgery may recover to a large degree, the relative micromotions between the implant and the surrounding tissue provoke chronic inflammatory responses, and mechanical mismatch between neural tissue and implantable neural probes can exacerbate those responses.^[^ ^77^, ^78^ ^]^ MEAs with flexible substrates can help to minimize the mechanical trauma caused by micromotion between the probe and the surrounding tissue.^[^ ^21^, ^79^ ^]^ The flexibility of a device is not only affected by the materials’ modulus but also by the geometry of the shank.^[^ ^21^ ^]^ Although polyimide has Young's modulus of ≈2 GPa, by making the film thin, the flexibility is increased so that MEAs can move with tissue to minimize relative micromotion.^[^ ^21^, ^80^ ^]^ For future development, emerging materials like fluorinated elastomers with modulus close to the neural tissue can be utilized as MEA substrates to further improve device integration.^[^ ^81^ ^]^

Qualitatively, MU appears to have worse tissue inflammation responses in the cortex than WT. The *Clock* gene is a known master gene that regulates a wide variety of inflammation pathways involving cytokine production, antioxidant response, and chemokine attraction.^[^ ^64^, ^65^ ^]^ More comprehensive and quantitative biochemical and histological studies will be performed in future studies to compare how the host tissue of MU and WT respond to implants. In summary, the electrophysiology data demonstrated that PEDOT/CNT coated MEAs have stable chronic recording performance during the 4‐week period.

### 3.5 The Relationship between MSN Activities and DA Dynamics

**Figure 10:**
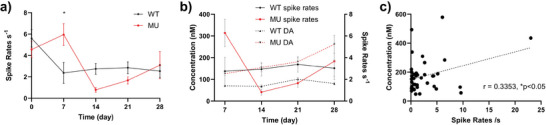
Spike rates of WT and MU over 4 weeks and its relationship with DA concentration dynamics. a). Change of spike rates over time in WT and MU. On day 7, MU had significantly higher spike rates than WT. (4 mice each group. Two‐way ANOVA with Šídák's multiple comparisons test. ^*^ *p*<0.5) b). Overlay plot of DA dynamics and spike rates (striatum region only) over the 4‐week period in WT and MU. Aside from day 7 from MU, it appears that spike rates and DA concentration were phase‐locked between day 14 and day 28 for both WT and MU. c). Scatter plot of spike rates vs detected DA concentration, DA and spike rates have a significant positive correlation. (Pearson correlation, r = 0.3353, n=43, ^*^ *p*<0.05)

The WT showed higher spike rates on day 0 than any later time points which may be the result of the acute stress induced by the traumatic surgical procedures (**Figure** **10**a).^[^ ^82^, ^83^, ^84^, ^85^ ^]^ The MU group showed significantly lower spike rates on day 7 than the WT, which could be a result of the *Clock* gene's effects on inflammation responses and tissue healing process (Figure 10a).^[^ ^13^, ^45^, ^84^, ^86^, ^87^, ^88^ ^]^ When overlaying the spike rates of the striatal regions from both WT and MU with the DA concentration changes over the 4‐week period, we observed a positive correlation between spike rates and DA concentration. WT's spike rate and DA concentration dynamics showed a very similar trend starting day 7 and MU's DA concentration profile appeared to be phase‐locked with spike rates starting day 14. When pulling all data points together in a scattered plot, a positive correlation is found between the DA concentration and the spike rate (Figure 10c, Pearson correlation, r = 0.3353, n = 43, ^*^ *p*<0.05). Considering that MSN receives DA input via D1 or D2 receptor and fire action potentials, this positive correlation is expected.^[^ ^8^, ^9^, ^10^, ^89^, ^90^ ^]^ However, MU had overall lower spike rates from day 14 to day 21 than WT while having a higher DA concentration (Figure 9b). It is possible that MSNs in MU are desensitized to DA input, resulting in the elevated DA concentration to compensate.^[^ ^91^, ^92^ ^]^

Previous work that has demonstrated successful in vivo application of dual‐mode flexible MEAs has been limited to acute study and amperometry DA detection that has low temporal resolution.^[^ ^33^ ^]^ Our previous work with PEDOT/CNT coated flexible GC MEAs showed successful SWV DA sensing for up to 4 weeks with low channel density (5 total channels) and no electrophysiology data.^[^ ^36^ ^]^ In the current work, PEDOT/CNT coated flexible MEAs with 20 channels demonstrated stable electrophysiology recording and SWV DA sensing over 4 weeks. This work demonstrates the great potential of dual‐function flexible MEAs in studying the coordinated effects of action potentials and neurotransmitters in facilitating behaviors and cognitive functions.

### 3.6 Limitations

The SWV scanning of DA had to be performed sequentially due to instrument limitations. Due to the temporal lag among different channels caused by sequential scanning, the DA sensing data was averaged over 5 min time bins which under‐utilized the temporal resolution of this technique. A multichannel potentiostat system that can simultaneously scan all channels will eliminate this problem.

The current work determines the sensitivity of each electrode using the current‐concentration relationship measured before implantation, which is called pre‐calibration. Due to the differences between PBS and brain tissue and potential protein adsorption and tissue encapsulation, using in vitro pre‐calibration may underestimate the in vivo concentration. However, previously we have found that the SWV current response of DA showed little drift measured at PEDOT/CNT‐functionalized CFEs in vivo between immediately upon probe implantation (less than 1 min) and up to 3 h after implantation, suggesting that the protein fouling is not a significant factor.^[^ ^35^ ^]^ By soaking the MEA in 20 mg/ml of albumin solution for 1 h, we found that the impedance of PEDOT/CNT was not significantly affected by protein adsorption (Figure S9, Supporting Information). The nanofibrous topography and the negative charges on CNTs may offer antifouling properties to the PEDOT/CNT coating. Another factor that could affect in vivo sensitivity is scar tissue encapsulation due to inflammatory response. Our previous work with PEDOT/CNT coating on glassy carbon flexible MEAs showed stable DA current response over the 4‐week period, indicating minimum sensitivity changes, thanks to the flexibility of the MEA substrate that minimizes scar tissue encapsulation.^[^ ^36^ ^]^ We also observed no degradation trend of DA current from the same electrode in this study. Taking together, the antifouling PEDOT/CNT coating and the flexible MEA help reduce the error of the absolute concentration estimation. In future studies, calibration methods may be improved. We have found in vitro that there is an inverse relationship between 25 Hz impedance and sensitivity (previous Figure 3e), now Figure S3b, Supporting Information). It may be possible to use this relationship to adjust the in vivo sensitivity based on the in vivo impedance. One approach is to use equivalent circuit modeling to define a circuit that accounts for the in vitro‐in vivo differences and apply such circuit components to the calibration measurement. This approach has been demonstrated to be effective for FSCV.^[^ ^93^ ^]^ Ultimately, in vivo calibration may provide a more accurate estimation but is very challenging to establish. This might be done by integrating a microfluidic channel on the sensor MEA with the outlet immediately next to the sensing electrode. A calibration curve may be obtained by injecting different known concentrations of DA in vivo. Unfortunately, it is impractical to calibrate every sensor this way in vivo, but we may use such a setup to validate and fine‐tune the impedance‐based calibration methods in future studies.

In this study, all in vivo measurements were done on anesthetized animals. It is well known that both DA dynamics and electrophysiology activities can be affected by ISO anesthesia. We took care to maintain the same level of ISO to the best of our ability. Future work will focus on overcoming challenges in utilizing this technique on free moving animals. This will reduce the cofounding effect from anesthetics and enable the acquisition of DA and electrophysiology data from free moving animals in various behavioral assays.

Overall, more channels of MEAs implanted in WT failed to detect DA than MU (Figure 7a,c). This is most likely a result of the intrinsically lower DA concentration in WT, which is approaching the detection limit of the sensor. For further work, the size of the electrode sites, PEDOT/CNT coating thickness and morphology can be further optimized to improve sensitivity.

## 4 Conclusion

In summary, we demonstrated the scalable and reproducible batch fabrication of dual‐function flexible MEAs for DA sensing and electrophysiology recording applications. The MEAs coated with organic polymer PEDOT/CNT can be utilized for stable long‐term DA sensing for up to 4‐weeks. For the first time, we demonstrated successful in vivo measurement of both tonic DA concentration and electrophysiology from the same electrode sites for a 4‐week period. The customized MEA channel layout also made simultaneous monitoring of both cortex and striatum regions possible. The implanted MEAs can generate both DA concentration profile and electrophysiology patterns with high spatial and temporal resolution than any analytic tools available. By comparing the measurements in MU to WT, we revealed that extracellular DA in the striatum is affected by the *Clock* gene.

This dual‐function flexible MEA can serve as a powerful tool to help better understand the underlying mechanisms of neurological processes and dysfunctions that involve the DA systems. Also, by optimizing the voltammetry waveform, the same device can also detect other electroactive neurotransmitters such as serotonin, providing a versatile platform that can be tailored toward different neuroscience applications.

## Conflict of Interest

The authors declare no conflict of interest.

## Author Contributions

Conceptualization: B.W, E.C, C.M and T.C.; methodology: B.W, E.C; validation: B.W; formal analysis: B.W; investigation: B.W., E.C, C.M and X.T.C; resources: C.M and X.T.C; data curation: B.W; writing—original draft preparation: B.W; writing—review and editing: B.W, C.M, and X.T.C.; supervision: C.M and X.T.C; project administration: B.W., C.M, and X.T.C; funding acquisition: C,M and X.T.C. All authors have read and agreed to the published version of the manuscript.

## Supporting information

Supporting Information

## Data Availability

The data that support the findings of this study are available from the corresponding author upon reasonable request.
