# Supplementary material for: PEDOT/CNT Flexible MEAs Reveal New Insights into the Clock Gene's Role in Dopamine Dynamics
Source: Adv Sci (Weinh). 2024 Mar 2;11(27):2308212. doi: 10.1002/advs.202308212 (PMC11251561; doi:10.1002/advs.202308212)
Supplement: Supplementary file 1 — Supporting Information [file ADVS-11-2308212-s001.pdf]

ADVANCED SCIENCE
Open Access

Supporting Information

for *Adv. Sci.*, DOI 10.1002/advs.202308212

PEDOT/CNT Flexible MEAs Reveal New Insights into the *Clock* Gene's Role in Dopamine Dynamics

*Bingchen Wu, Elisa Castagnola, Colleen A. McClung and Xinyan Tracy Cui**

PEDOT/CNT flexible MEAs reveal new insights into the Clock gene's role in dopamine dynamics

Bingchen Wu[1,2], Elisa Castagnola[1,3], Colleen McClung[4], Xinyan Tracy Cui[1,2,5 #]

1. Department of Bioengineering, University of Pittsburgh. Pittsburgh, PA ,15213, USA.
2. Center for the Neural Basis of Cognition, Pittsburgh, PA, 15213, USA.
3. Department of Biomedical Engineering, Louisiana Tech University, Ruston, LA 71272, USA.
4. Department of Psychiatry, University of Pittsburgh. Pittsburgh, PA ,15213, USA.
5. McGowan Institute for Regenerative Medicine, Pittsburgh, PA 15219, USA

# Corresponding Author: xic11@pitt.edu

Fitted line	Week (t)	Slope (S)	Y-intersect (b)
Y = 0.1756*X - 12.02	1w	0.1756	12.02
Y = 0.07687*X - 3.672	2w	0.07687	3.672
Y = 0.08603*X - 6.742	3w	0.08603	6.742
Y = 0.06559*X - 3.587	4w	0.06559	3.587

Peak current: I
Slope of specific week: S_t
Y-intersect of specific week: b_t
Dopamine concentration: [DA]

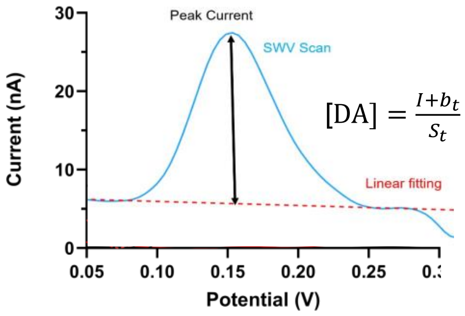

Figure S1. Calibration curve table for equation, slope, and Y-intersect of each time point. Demonstration of SWV DA redox peak extraction and equation for DA concentration conversion.

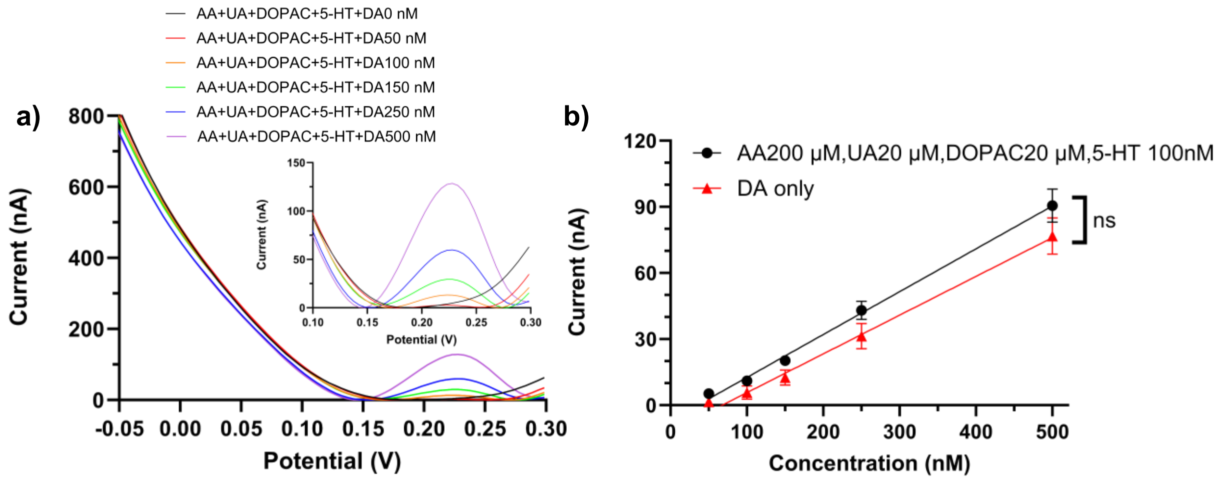

Figure S2. Selectivity test of PEODT/CNT coated flexible MEAs in the presence of interfering compounds at physiologically relevant range. a). SWV waveforms at different DA concentrations in the presence of interfering compounds. Inset shows DA redox current response within the DA specific potential window (0.1-0.3V). b). Calibration curves comparison with or without interfering compounds. n=20. No significant difference in sensitivity is observed.

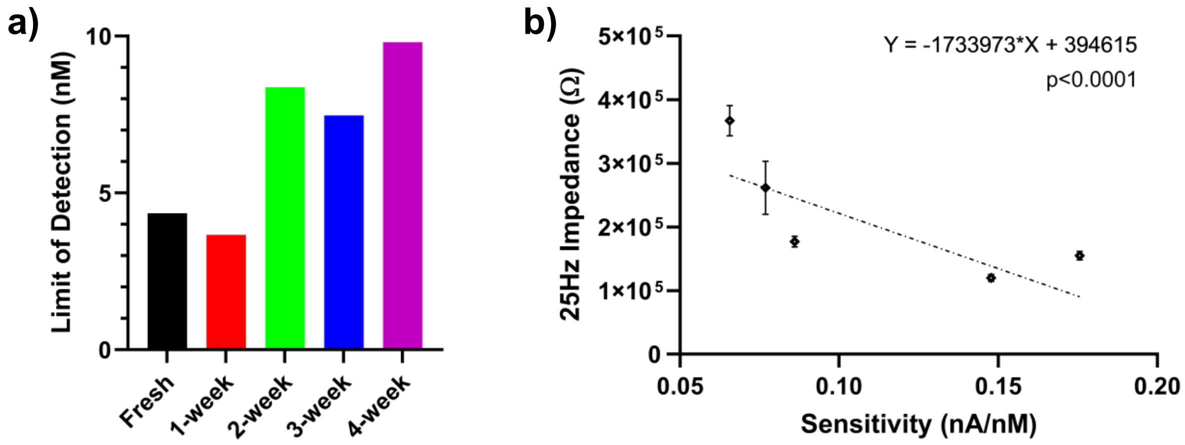

Figure S3. a). Limit of Detection of PEODT/CNT coated flexible MEAs from 4-week In vitro stability test. b). 25Hz impedance vs sensitivity. The impedance and sensitivity are negatively correlated. (Simple liner regression, p<0.0001)

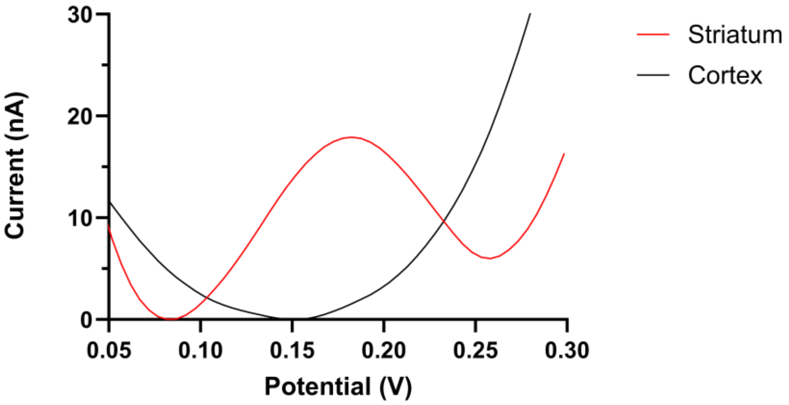

Figure S4. Representative SWV waveform of sites in cortex vs in striatum. Cortex sites don't have DA redox peaks around 0.18V.

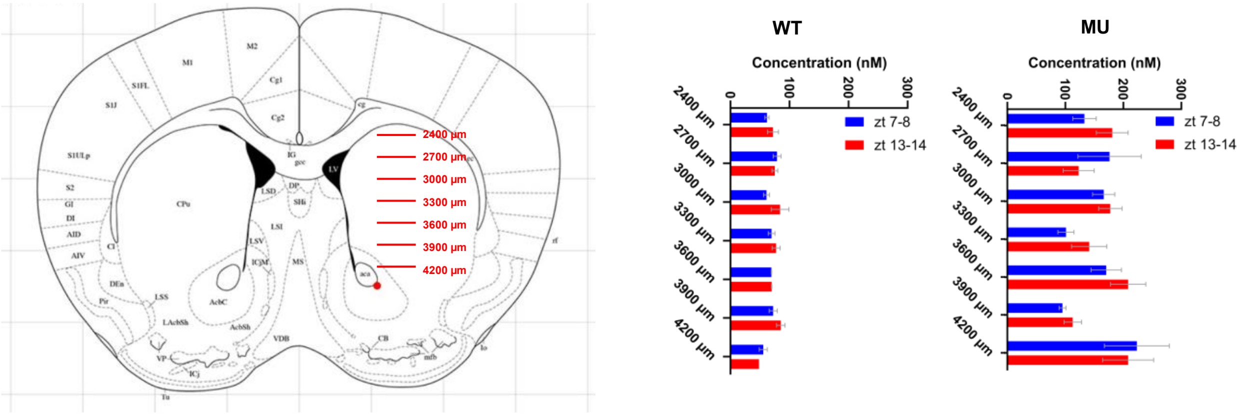

Figure S5. Spatial patterns of DA concentrations for ZT 7-8 and ZT 13-14 in WT and MU with corresponding locations in the striatum. Each depth labeled red line in the atlas corresponds to the same depth in the bar plot. The red dot indicates the tip of the MEA shank.

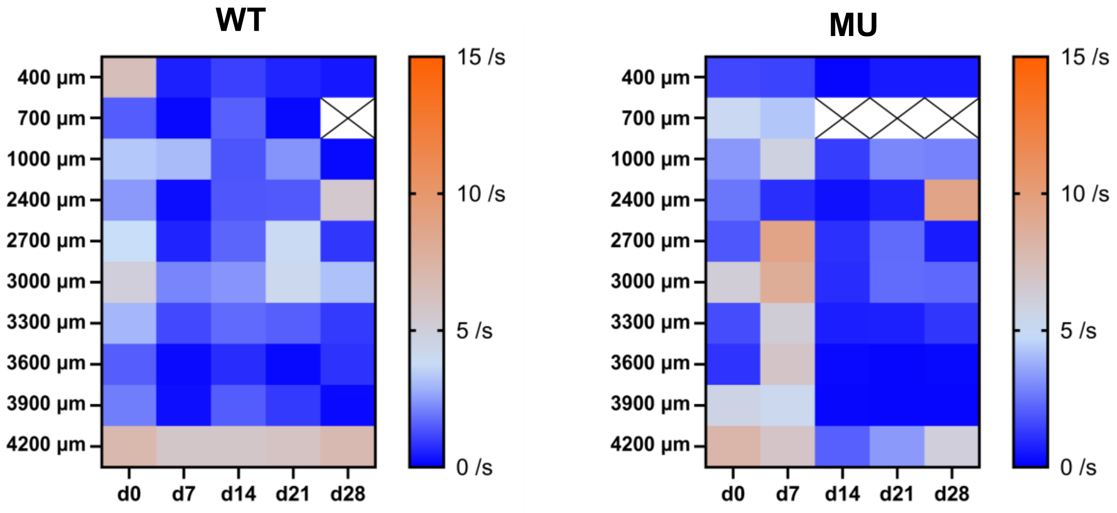

Figure S6. Heatmap of spatial and temporal distribution of spike rates in WT and MU over 4 weeks.

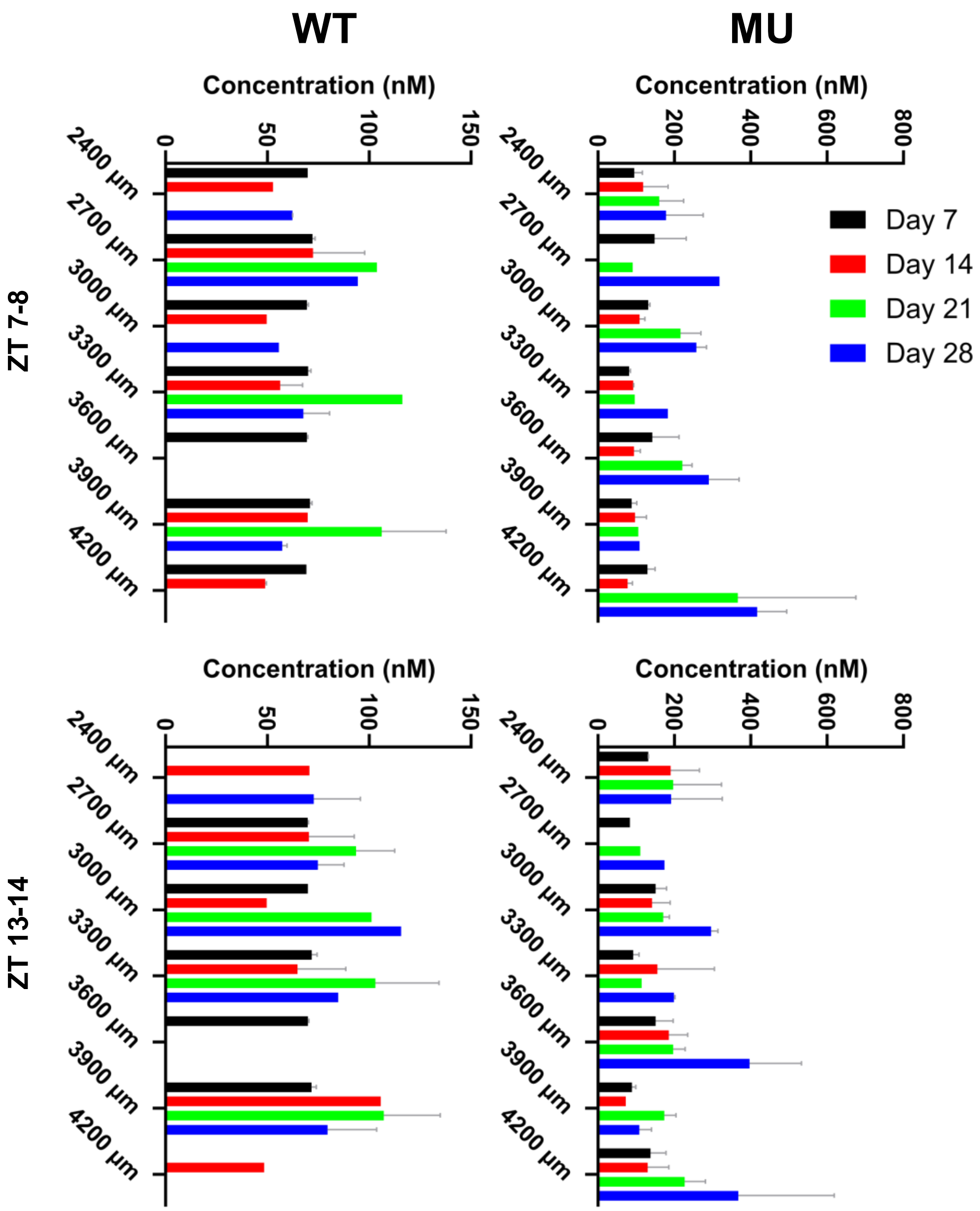

Fig S7. Sensor-to-sensor variations across all animal groups over 4-week time periods. Values are shown as mean±sd. Missing values indicate no data were collected on that depth or time. Missing error bars indicate only one animal has data collected for the depth or time.

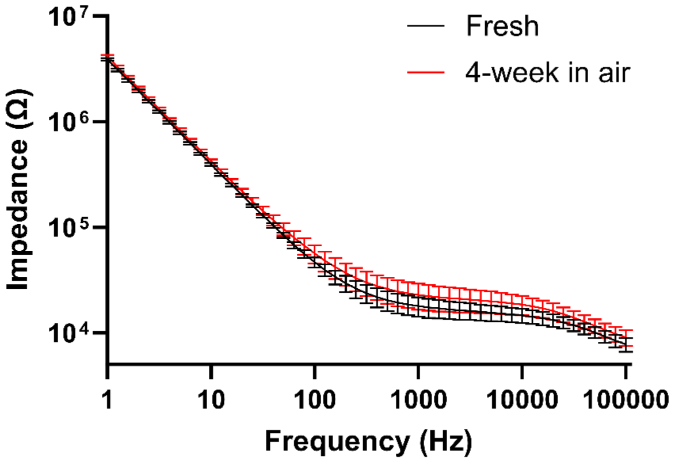

Figure S8. EIS measurements of PEDOT/CNT coated MEAs after 4 weeks dry stored. No changes observed. n = 20. Mean ± SD.

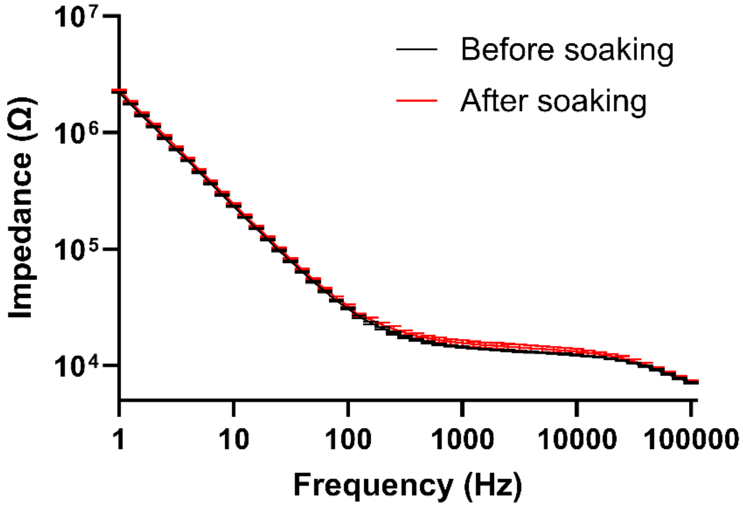

Figure S9. EIS measurements of PEDOT/CNT coated MEAs before and after soaked in albumin solution for 1 hr (20 mg/ml). n=16.
